# The Valproate Mediates Radio-Bidirectional Regulation Through RFWD3-Dependent Ubiquitination on Rad51

**DOI:** 10.3389/fonc.2021.646256

**Published:** 2021-03-25

**Authors:** Guochao Liu, David Lim, Zuchao Cai, Wenwen Ding, Zhujun Tian, Chao Dong, Fengmei Zhang, Gongshe Guo, Xiaowei Wang, Pingkun Zhou, Zhihui Feng

**Affiliations:** ^1^ Department of Occupational Health and Occupational Medicine, School of Public Health, Cheeloo College of Medicine, Shandong University, Jinan, China; ^2^ School of Health Sciences, Western Sydney University, Campbelltown, NSW, Australia; ^3^ College of Medicine and Public Health, Flinders University, Bedford Park, SA, Australia; ^4^ School of Biomedical Sciences, Li Ka Shing Faculty of Medicine, The University of Hong Kong, Hong Kong, China; ^5^ Department of Medicine, The Second Hospital, Cheeloo College of Medicine, Shandong University, Jinan, China; ^6^ Department of Radiation Oncology, Washington University, School of Medicine, St. Louis, MO, United States; ^7^ Beijing Key Laboratory for Radiobiology, Department of Radiation Biology, Beijing Institute of Radiation Medicine, AMMS, Beijing, China

**Keywords:** valproate, radiosensitization, radioprotection, ubiquitination, RFWD3, Rad51

## Abstract

Ionizing radiation (IR) can induce DNA double-strand breaks (DSBs) in tumor cells during radiotherapy (RT), but the efficiency of RT is limited because of the toxicity to normal cells. Locating an adjuvant treatment to alleviate damage in normal cells while sensitizing tumor cells to IR has attracted much attention. Here, using the 7,12-dimethylbenz[α]anthracene (DMBA)-induced malignant transformed MCF10A cells, we found that valproate (VPA), a histone deacetylase inhibitor (HDACi), radiosensitized transformed cells while alleviated IR-induced damage in normal cells at a safe dose (0.5 mM). We further demonstrated the decrease of homologous recombination (HR)-associated Rad51 in the transformed cells was related to the increase of its ubiquitination regulated by E3 ligase RFWD3 for the radiosensitization, which was opposite to normal cells, indicating that RFWD3-dependent ubiquitination on Rad51 was involved in the VPA-mediated radio-bidirectional effect. Through DMBA-transformed breast cancer rat model, VPA at 200 mg/kg radiosensitized tumor tissue cells by increasing RFWD3 and inhibited Rad51, while radioprotected normal tissue cells by decreasing RFWD3 and enhanced Rad51. In addition, we found high-level Rad51 was associated with tumorigenesis and poor prognosis in breast cancer patients. Our findings uncovered RFWD3-dependent Rad51 ubiquitination was the novel mechanism of VPA-mediated radio-bidirectional effect, VPA is a potential adjuvant treatment for tumor RT.

## Introduction

Radiotherapy (RT) has been proven effective in managing breast cancer and other malignancies (1), but the toxicity and the bystander adverse effect induced by ionizing radiation (IR) have limited the popularity of this oncological treatment modality (2–4). Furthermore, multiple tumor cells have shown resistance to RT (5). Therefore, locating an efficacious and safe oncological treatment modality is of clinical importance.

IR induces intracellular DNA damage, particularly the most lethal DNA double-strand breaks (DSBs), and triggers sophisticated DNA repair pathways, including error-free homologous recombination (HR) and error-prone nonhomologous end-joining (NHEJ) (6). HR acts as an effective DSBs repair pathway to keep genomic stability (5). Rad51 has been identified as the central protein in HR, which forms helical nucleoprotein filaments on tracts of single-strand DNA (ssDNA) at DSBs sites (7). Rad51 also regulates stalled replication forks by protecting newly synthesized DNA from degradation (8, 9), promoting replication fork regression (9, 10), and reinstates the collapsed replication forks (11).

As the DNA damage response (DDR) is initiated, DNA repair proteins are recruited to damaged sites to repair DSBs. A cascade of post-transcriptional modification (PTM) associated protein interactions occur at the damaged sites, which serve as the codes of DNA damage to recruiting specific proteins for repair (12). DDR is exquisitely regulated by the modification of numerous proteins, ubiquitination plays an important role (13), which has been demonstrated to span a wide spectrum in many cellular functions, including proteasomal degradation of proteins, intracellular trafficking, inflammation signaling, and DNA repair (14, 15). The deregulation of ubiquitination could also result in oncogenesis and the dysfunction of the DNA repair process (16, 17). Notably, the ubiquitination on Rad51 is suggested as an important way to maintain HR function (18). RFWD3, a novel E3 ligase, was found to be necessary for the restart of the replication fork during the HR process (18, 19). Current studies demonstrated that Rad51 can be ubiquitinated by RFWD3, which is necessary for the restart of the replication fork during the HR process (18). Rad51 directly interacts with the N-terminal of RFWD3 and is ubiquitinated by RFWD3 (20, 21). Also, it is reported the loss of the ubiquitination of Rad51 leads to impaired HR (18). Since studies demonstrated that DDR proteins may be potentially targeted for cancer therapy (22, 23), thus, it would be important to locate a drug that can specially target RFWD3-dependent Rad51 ubiquitination for cancer therapy.

The anticonvulsant drug, valproate (VPA) is a class IIa histone deacetylase inhibitor (HDACi) (24–27), it has been demonstrated to sensitize multiple tumor cells to IR treatment, increased intracellular toxicity, and a safe and effective radiosensitizer following IR treatment. The radiosensitization effect of HDACi was further found in glioma, lung cancer, esophagus cancer, prostatic cancer, and colon cancer (24, 28–32). Our previous studies showed that VPA, at a safe therapeutic dose of 0.5 mM, sensitizes breast tumor MCF7 cells and the primary cultured breast tumor cells to IR by impeding HR function with Rad51 inhibition (33). Recent studies have shown that VPA protects normal neuron cells under IR treatment through regulating apoptosis, reactive oxygen species (ROS) response, and Nrf2 translocation (31, 32, 34). The lethality of mice after total-body irradiation was mitigated by concomitant VPA treatment (35). Moreover, VPA reduced hair loss and improved survival in high-grade glioma patients during RT (36). Although the potential of VPA for being an effective radioprotectant and radiosensitizer on normal neuron and glioma cells has been demonstrated, the accurate mechanism of this bidirectional effect of VPA upon IR-induced damage on normal and malignant cells is still unclear.

In this study, we used the environmental carcinogen 7,12-dimethylbenz[α]anthracene (DMBA) to establish a transformed malignant cell from the human normal breast epithelial cell line MCF10A. By this paired cell line, we found that VPA renders radiosensitization effect on DMBA-transformed MCF10A cells while the radioprotective effects on normal MCF10A. Importantly, VPA-mediated radiosensitization effects on transformed cells were associated with the up-regulation of Rad51 ubiquitination mediated by RFWD3. In contrast, VPA-mediated radioprotective effects on normal cells were due to Rad51 ubiquitination down-regulated by RFWD3. Using a well-established breast cancer rat model induced by DMBA, the RFWD3-dependent Rad51 ubiquitination involved in the VPA-mediated bidirectional effect under RT was also confirmed. Our findings uncovered the molecular mechanism of the bidirectional effect of VPA under IR treatment both *in vitro* and *in vivo*, VPA would be able to target RFWD3 and trigger Rad51 ubiquitination for cancer radiotherapy.

## Materials and Methods

### Ionizing Radiation

IR was applied to rats and cells by using a Siemens Stabilipan 2X-ray generator at 250 kVp 12 mA, at a dose rate of 2.08 Gy/min. The schematic diagram of local irradiation on tumors of rats is shown and described in **Supplementary Figure 4B**.

### VPA Preparation and Treatment

Sodium VPA (P4543, Sigma, St. Louis, MO, USA) was diluted with the cell culture medium to 0.5 mM. VPA in the culture medium was added to cells 24 h prior to IR treatment. For rats, sodium VPA was dissolved in sterile saline and injected into the peritoneum.

### Cell Culture and Transfection

The MCF10A cell line was provided by Stem Cell Bank, Chinese Academy of Sciences, cultured with DMEM/F12 (D9785, Sigma) and combined with growth factors (37). MCF10A cells were treated with DMBA at different doses for 24 h then cultured for around 60 days with fresh medium for transformation. RI-1 (S8077, Selleckchem, Houston, TX, USA) was dissolved with DMSO, cells treated with 10 μm RI-1 in culture medium for 24 h post IR treatment. 16HBE and HCC1937 cell lines were maintained in DMEM with 10% fetal bovine serum (FBS, 16140071, Gibco, Thermo Fisher, Waltham, MA, USA). HCC1937 cells were transfected with expression vectors containing wild-type BRCA1 (pcDNA3-BRCA1, wtBRCA1). siRFWD3 [siRFWD3-1: GGACCUACUUGCAAACUAUdTdT; siRFWD3-2: GCAGUCAUGUGCAGGAGUUdTdT (18, 38)] was transfected with lipofectamine 2000 (12566014, Thermo Fisher) in accordance with the manufacturer’s instructions.

### Inhibition of Apoptosis

Z-VAD-FMK (HY-16658B, MedChemExpress, Monmouth Junction, NJ, USA) was dissolved in DMSO to 1 mM for stocking and further diluted by culture medium to 40 μm for a working solution. Cells were treated by Z-VAD-FMK for 2 h prior to VPA and IR treatment.

### Comet Assay

The neutral and alkaline comet assay were performed on the Trevigen Comet Assay kit (Cat. 4252-040-K, Trevigen, Gaithersburg, Maryland, USA). Cells were pretreated with 0.5 mM VPA for 24 h followed by 8 Gy IR treatment. Low melting agar was combined with cell suspension at a ratio of 1:10 after treatments, then spread onto comet slide immediately. The slides were cooled for 30 min, then immersed in lysis solution. Slides were immersed into cold alkaline unwinding solution for 1 h (alkaline comet), then transferred into neutral or alkaline electrophoresis buffer at 21 V for 30 min at 4°C in the dark. The slides were washed twice in distilled water and 70% ethanol for 5 min, then dried and stained with SYBR gold (1:10000, Trevigen).

### Immunofluorescence

This protocol was described in our previous studies (39). Primary antibodies included: γH2AX (1:500; 05-636, EMD Millipore, Burlington, MA, USA), Rad51 (1:100; sc8349, Santa Cruz, Dallas, TX, USA), BRCA1 (1:100; sc-6954, Santa Cruz), and 53BP1(1:1000; NB100-304, Gene Tex, Irvine, CA, USA), RFWD3 (1:200; 19893-1-AP; Proteintech, Wuhan, China). Secondary antibodies consisted of Alexa Fluor® 594 goat anti-mouse lgG(H+L) (1:300; A11032, Molecular probes, Waltham, MA, USA) and Alexa Fluor® 488 chicken anti-rabbit lgG(H+L) (1:300; A21441, Molecular probes, Thermo Fisher).

### Western Blotting

The protocol was described in our previous publication (40). Primary antibodies included: BRCA1 (1:200; sc-6954, Santa Cruz), Rad51 (1:200; sc-8349, Santa Cruz), Rad51 (1:2000; PC130, Calbiochem, Burlington, MA, USA), P53 (1:200; sc-98, Santa Cruz), P21 (1:150; 556431; BD, New York, NY, USA), RFWD3 (1:500; 19893-1-AP; Proteintech, Wuhan, China), Ubiquitin (1:1000; 3936s; Cell Signaling Technology, Trask Lane Danvers, MA, USA), β-actin (1:2000; TA-09, ZSGB-BIO, Beijing, China) and GAPDH (1:2000; TA-08, ZSGB-BIO). Secondary antibodies were goat anti-mouse lgG (H+L) (1:5000; 31430, Thermo Fisher) and goat anti-rabbit lgG (H+L) (1:5000; 31460, Thermo Fisher).

### Immunoprecipitation

Whole-cell lysate was mixed with Rad51 antibody (40ul/1mg protein lysate; PC130, Calbiochem) at 4°C for 3 h, protein-A dynabeads were added to the lysate-antibody mixture at 4°C overnight. The mixture was washed four times and then denatured at 95°C for 5 min. The subsequent steps were the same as the regular Western blot described above.

### Clonogenic Survival and Serum-Starving Assay

The method of clonogenic survival assay was described elsewhere (41). For the serum-starving assay, we used cells at the number of 1 × 10^5^ cultured with medium containing or lacking horse serum. The medium without serum still contained the necessary factors for maintaining the proliferation of MCF10A cells.

### Soft Agar Colony Formation Assay

For the bottom layer, 1.5 ml/well 0.6% low-melting agar (214230, Difco Agar Nobel, BD) was added to a six-well plate for solidification. The top layer contained cell suspension (1 × 10^4^/well) and 0.3% agar. The cells were cultured for 3 to 4 weeks until a visible colony formed; these were stained with 0.005% crystal violet (C8470, Solarbio, Beijing, China).

### Animal Keeping and the Establishment of Breast Cancer Model

Detailed information was described in our previous publication (33). The experiment was performed in accordance with the requirements of the Shandong University Human and Animal Ethics Research Committee (81472800, approved March 2014). In brief, around 40 to 60 days after a single dose of 20 mg DMBA gavage to female Sprague Dawley (SD) rats, lumps in breast sites could be found by palpation (33).

### The Detection of VPA Concentration in Serum

Peripheral blood was collected from the jugular vein of the SD rats at 15 min, 1 h, 3 h, 6 h, 12 h and 24 h after VPA treatment. The concentration of VPA in serum was detected by Emit kit (Emit® 2000 VPA detection kit; Siemens).

### Tissue Array Analysis

Tissue arrays were purchased from Shanghai Outdo Biotech Company (Shanghai, China), which collected 140 breast cancer tissues and 45 breast para-carcinoma tissues obtained from different breast cancer patients admitted to the public hospitals in southern China between 2004 and 2014 and approved by the Human Research Ethics Committee (HREC) of Taizhou Hospital of Zhejiang Province, informed consent was signed by all the patients involved in this study. Immunohistochemistry staining of Rad51 in tissues was performed and the density (H-score) of Rad51 expression in the tissues was calculated. Since the expression of Rad51 in para-carcinoma tissues did not follow a normal distribution, the median (50%) expression of Rad51 in para-carcinoma was used to define tumor tissue as of lower (n = 117) and higher (n = 20) expression. Survival data of the patients 120 months post the sample biopsy was obtained.

### Statistical Analysis

Results were presented as means ± standard deviation for the groups. Data were analyzed by independent sample t-test and further normalized by Bonferroni post hoc test, survival data in tissue arrays were analyzed by Kaplan-Meier survival analysis on SPSS Statistics for Windows, Version 23.0 (Armonk, NY: IBM Corp; licensed to Shandong University). *P* < 0.05 indicated a statistically significant difference.

## Results

### The Establishment of a DMBA-Induced Highly Malignant Transformation Cell Model on Normal Cell MCF10A

**Figure 1:**
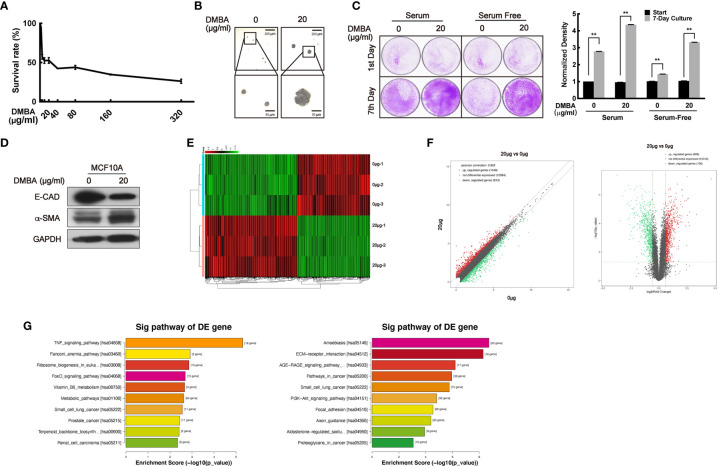
The establishment of a DMBA-induced malignant transformation cell model on normal cell MCF10A. **(A)** MTT assay was performed for the toxicity detection of DMBA on MCF10A. **(B)** Soft agar assay showed the forming colonies after 4 weeks of culturing to identify cell transforming. **(C)** Cells were cultured under different serum conditions to detect their growth ability to identify cell transforming. **(D)** The expression of E-CAD and α-SMA was detected by Western blot both on 0 and 20 μg/ml DMBA-treated MCF10A cells. **(E)** The heat map from RNA sequencing analysis showed the differentially expressed genes between 20 and 0 μg/ml DMBA-treated cells. **(F)** Scatter plot (left) and volcano plot (right) exhibited the changed-genes between the two cell lines. **(G)** Genes were analyzed by KEGG database for clustering functional pathways, enrichment score was used as the measurements. Each data point in the graph was from three independent experiments (mean ± SD); *P*-values were calculated by t-test (**P < 0.01).

To confirm the bidirectional effect of VPA on tumor and normal cells, we sought to transform normal MCF10A cells to malignancy by DMBA treatment and establish a paired cell line. First, a suitable dose of DMBA treatment on MCF10A cells was explored through MTT assay. The doses of DMBA over 80 μg/ml exhibited increasing cytotoxicity (**Figure 1A**), so doses less than 80 μg/ml DMBA were chosen to treat the normal MCF10A cell for 24 h and further cultured for around 60 days. Compared with the normal cells, 20 μg/ml DMBA-treated cells exhibited stronger ability to form colonies on the soft agar-colony formation assay (**Figure 1B**), demonstrated increased proliferating ability on the cell clonogenic assay (**Figure 1C**, *P* < 0.01), decreased E-CAD protein levels and increased α-SMA protein levels (**Figure 1D**), thus suggesting that DMBA was able to cause malignant transformation of normal cells (42–44). To verify this paired cell line, we next performed RNA sequencing analysis to detect the differential gene expression (**Figure 1E**). We found 909 up-regulated genes and 726 down-regulated genes in the DMBA-treated cells as compared with normal cells (**Figure 1F**). KEGG pathway analysis further indicated that the changed-genes were highly associated with breast cancer and other cancer (small cell lung cancer, prostate cancer, and renal cell carcinoma) pathways (**Figure 1G**, left panel: up-regulated, *P* < 0.05; right panel: down-regulated, *P* < 0.05). Our data demonstrated that 20 μg/ml DMBA resulted in MCF10A cell transformation, and a stabilized DMBA-induced malignant transforming cell model was successfully established.

### VPA Sensitizes Transformed cells While Protecting Normal Cells After IR Treatment by Regulating the Rad51-Mediated HR Pathway

To investigate the effect of VPA on both the DMBA-induced transformed cells and normal cells after IR treatment, we next treated the cells with 0.5 mM VPA for 24 h prior to IR.

**Figure 2:**
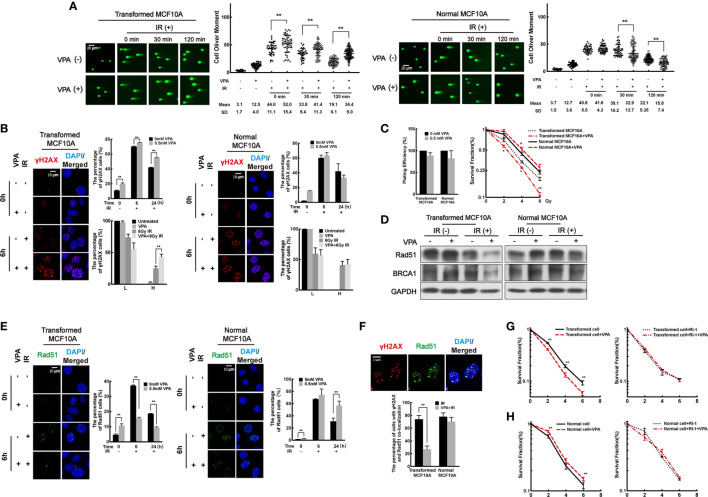
VPA sensitizes transformed cells while protects normal cells after IR treatment by regulating the Rad51-mediated HR pathway. **(A)** Transformed cells (left panel) and normal cells (right panel) were pretreated with VPA for 24 h and then subjected to 8 Gy IR. The DSB levels of cells at the indicated time points post-IR were detected by neutral comet assay. **(B)** Immunofluorescence staining of γH2AX foci at 6 and 24 h post-IR. The percentage of L-type and H-type cells containing γH2AX foci was showed in the graph. **(C)** The survival of transformed and normal cells was detected by colony formation assay. **(D)** At 6 h post-IR, transformed and normal MCF10A cells were subjected to Western blotting. **(E)** Rad51 foci formation by immunofluorescence. **(F)** At 6 h post-IR, the percentage of γH2AX and Rad51 colocalized cells were showed in the graph. The colony formation and graph showed the cell survival both in **(G)** transformed and **(H)** normal cells under RI-1 inhibition on Rad51. Each data point in the graph was from three independent experiments (mean ± SD); *P*-values were calculated by t-test (*P < 0.05, **P < 0.01).

First, DSB levels were measured in the paired cell line. By neutral comet assay, we found that DSB levels in the VPA-treated DMBA-transformed cells were increased at 0 min, 30 min, and 120 min post-IR (**Figure 2A**, left panel; *P*<0.01). However, in the untransformed normal cells, decreased DSB levels were observed in the combination group at 30 min and 120 min post-IR (**Figure 2A**, right panel; *P* < 0.01). The results were validated by the alkaline comet assay (**Supplementary Figure 1A**). To further detect the DSB levels in the cells, we next explored the foci formation of DSBs markers, γH2AX and 53BP1, by immunofluorescence. High levels of DSBs were detected at 6 h post-IR in both transformed and normal cells (**Figure 2B**, **Supplementary Figure 1B**). Cells with γH2AX or 53BP1 foci were divided into two groups at 6 h post-IR treatment: lower (L) type (under 20 foci per cell) and higher (H)-type (over 20 foci per cell). In the transformed cell, VPA enhanced the H-type cells at 6 h post-IR treatment (*P* < 0.01), and there were persistent γH2AX and 53BP1 foci cells with concomitant VPA treatment at 24 h post-IR (*P* < 0.01, **Figure 2B** left panel, **Supplementary Figure 1B** left panel). In contrast, in the normal cells, there was no significant difference in the percentage with L-type and H-type cells between the groups. Noticeably, VPA attenuated the γH2AX or 53BP1 foci cells at 24 h post-IR treatment (**Figure 2B** right panel, **Supplementary Figure 1B** right panel). These results demonstrated an elevated DSBs in the transformed cell line as compared to decreased DSBs levels in the normal cell line with VPA neoadjuvant treatment before IR.

Next, cell survival was measured in both cell lines by clonogenic assay. We observed that VPA compromised the survival of transformed cells but promoted the survival of normal cells with IR treatment (**Figure 2C**). Similarly, VPA also promoted the survival of human bronchial epithelial cell line, 16HBE, in response to IR treatment (**Supplementary Figure 1C**), thus confirming the effect of VPA on normal cells was distinct to the DMBA-transformed malignant cells. Collectively, our results indicated that VPA renders radiosensitization on DMBA-induced transformed cells and radioprotection on normal cells.

To examine whether VPA-mediated dual-role effect was associated with DNA repair function in both cell lines, we measured the protein level and foci formation of key proteins in the NHEJ and HR pathways. We found the protein level of NHEJ key proteins, including Ku70/80 and DNA-PKcs, were not noticeably affected by VPA treatment in our working system (**Supplementary Figure 1D**), indicating that NHEJ pathway was not significantly affected by VPA treatment in this working system. However, we observed that VPA inhibited the protein level of Rad51 and BRCA1 of the HR pathway in the transformed cells with IR treatment (**Figure 2D**). Meanwhile, to confirm the effect of VPA on malignant cells, we also tested the expression of Rad51 and BRCA1 in several breast cancer cell lines including MCF7, MDA-MB-231, and EUFA423. VPA inhibited the protein expression of Rad51 and BRCA1 upon IR treatment in those breast cancer cell lines (**Supplementary Figure 1E**, upper panel), which was consistent with the finding in the transformed cells. In contrast, in the normal MCF10A cells, the protein expression of Rad51 and BRCA1 were not noticeably affected by VPA and IR treatment (**Figure 2D**) and were found to be increased in the 16HBE cells (**Supplementary Figure 1E**, lower panel). The percentage of cells containing Rad51 and BRCA1 foci was reduced by VPA in the transformed cells and was increased in the normal cells following VPA and IR treatment (**Figure 2E**, **Supplementary Figure 1F**). We also observed that VPA decreased the number of cells with Rad51 and γH2AX foci colocalization after IR treatment in the transformed cells at 6 h post-IR treatment (*P* < 0.01), but not in the normal cells (**Figure 2F**). These results indicated that the Rad51-mediated HR function was inhibited by VPA in the transformed cell lines, while it was preserved in the normal cell lines upon IR treatment.

To investigate whether the VPA-mediated bidirectional effect on transformed and normal cells is Rad51-dependent, the Rad51 inhibitor, RI-1, which covalently binds to the cysteine 319 on the surface of Rad51 protein for destabilization (45), was employed for the next series of study. A 10 μM RI-1 can significantly inhibit Rad51 protein expression in both transformed and normal MCF10A cells (**Supplementary Figure 2A**). Rad51 inhibition leads to a more sensitive phenotype to IR treatment and compromised the radiosensitization effect of VPA (**Figure 2G**). However, in the normal MCF10A cells, Rad51 inhibition also compromised this protection effect under IR treatment (**Figure 2H**), which was further validated by the 16HBE cell line (**Supplementary Figure 2B**). We also confirmed that BRCA1 played a similar effect as Rad51 using an isogenic paired cell line of HCC1937 with and without BRCA1 expression, which demonstrated the radiosensitization effect of VPA was compromised by BRCA1-deficiency (**Supplementary Figure 2C**).

The above results demonstrated that the bidirectional effect of VPA was associated with Rad51-mediated HR function. VPA preserved HR through regulating BRCA1-Rad51 for protecting normal cells under IR treatment, while disrupted HR function leading to destroying transformed cells.

### VPA Affects Rad51 Stability and Ubiquitination During the Bidirectional Process

**Figure 3:**
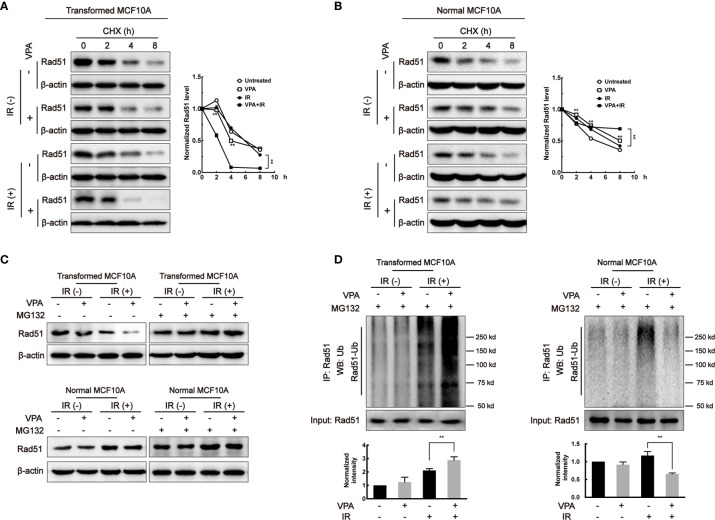
VPA affects Rad51 stability and ubiquitination. After VPA and IR treatments, **(A)** transformed MCF10A cells and **(B)** normal MCF10A cells treated with 40 μg/ml CHX for 0, 2, 4, and 8 h. The expression of Rad51 was detected by Western blot. Cells treated with 40 μM MG132 at 6 h before whole-cell extracts harvesting. **(C)** Rad51 protein expression was detected by Western blot and the **(D)** ubiquitination on Rad51 was detected by immunoprecipitation. Bands in the graphs were quantified by ImageJ software (Wayne Rasband). Each data point in the graphs was from three independent experiments (mean ± SD); *P*-values were calculated by t-test (**P < 0.01).

To explore whether the change in Rad51 protein level was associated with its protein stability, cycloheximide (CHX), a protein synthesis inhibitor, was added to the paired cells and Rad51 protein level was subjected to western blot. In the transformed cell, the Rad51 half-life was shortened in the VPA alone (4.08 h, *P* < 0.01) and combination treatment (2.42 h, *P* < 0.01) groups as compared with untreated (6.06 h) and IR (5.90 h) groups (**Figure 3A**). In contrast, in the MCF10A normal cells, we found the half-life of Rad51 protein was significantly prolonged by VPA treatment both in the VPA alone (8.07 h, *P* < 0.01) and combination treatment (>8 h, *P* < 0.01) group as compared with untreated (4.93 h) and IR (6.82 h) groups (**Figure 3B**). Similar results were detected with BRCA1 protein in the transformed cells (untreated, 8.04 h; VPA, 3.30 h; IR, 6.75 h; VPA+IR, 3.18 h; **Supplementary Figure 3A**) and normal cells (untreated, 5.54 h; VPA, 7.11 h; IR, 3.42 h; VPA+IR, 8.06 h; **Supplementary Figure 3B**).

Since we discovered that the Rad51 protein stability was related to the VPA-mediated bidirectional effect, it is necessary to detect its ubiquitination in the paired cell line. We used the 26S proteasome inhibitor, MG132 to treat cells for 6 h before harvesting. **Figure 3C** showed the effect of VPA on Rad51 protein levels in both the transformed and normal cells were restored by MG132. We found that the ubiquitination of Rad51 was increased in transformed cells and decreased in normal cells by the combined treatment as compared to IR alone (*P*<0.01, **Figure 3D**). Our data suggested that VPAs exhibited a bidirectional role in transformed and normal cells by regulating Rad51 ubiquitination.

### RFWD3-Dependent Rad51 Ubiquitination Is Involved in the Observed VPA-Mediated Bidirectional Effect

**Figure 4:**
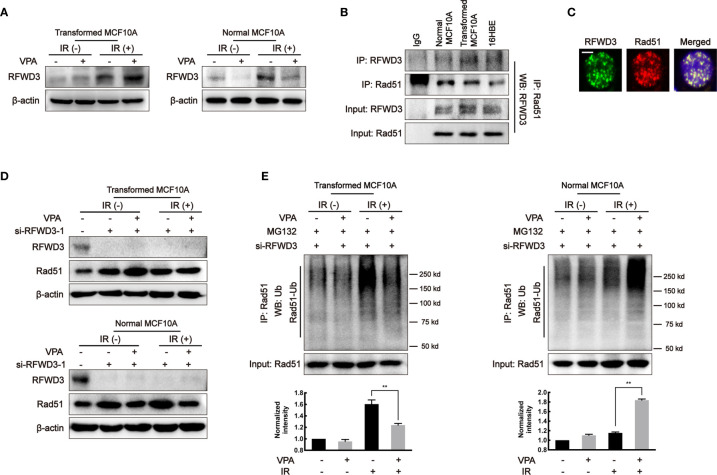
RFWD3-dependent Rad51 ubiquitination is involved in the VPA-mediated bidirectional effect. **(A)** RFWD3 protein expression was detected with VPA and IR treatment. **(B)** The interaction between RFWD3 and Rad51 proteins was detected by protein beads containing Rad51 antibody through Western blot. **(C)** The foci formation of RFWD3 and Rad51 in this paired cell was detected at 6 h post-8 Gy IR. Co-localization of RFWD3 and Rad51 proteins was shown in the figure. The scale bar represented as 10µm. **(D)** siRNA was designed to abolish the function of RFWD3 and the expression of Rad51 under those treatments was detected by Western blot. **(E)** Ubiquitination of Rad51 on transformed and normal MCF10A cells after RFWD3 depletion was detected by Western blot. Bands in the graphs were quantified by ImageJ software (Wayne Rasband). Each data point in the graph was from three independent experiments (mean ± SD); *P*-values were calculated by t-test (**P < 0.01).

It was reported that E3 ligase, RFWD3, can directly interact and ubiquitinate Rad51, thus the effect of RFWD3 was investigated in our working system. In the transformed cells, the protein level of RFWD3 in the VPA+IR combination group was increased as compared with IR alone; in contrast, the combined treatment of VPA and IR decreased RFWD3 expression compared with IR alone in the normal MCF10A cells (**Figure 4A**). The change of RFWD3 in the combination group was confirmed in MCF7, MDA-MB-231, and EUFA423 breast cancer cell lines and normal 16HBE cell line (**Supplementary Figure 3C**). The protein expression was increased in cancer cells and decreased in normal cells, indicating that the VPA-mediated bidirectional effect was associated with RFWD3. We next found that both RFWD3 and Rad51 were in a protein complex by co-immunoprecipitation experiment in the three cell lines (**Figure 4B**), and both protein foci formation co-localized together after IR treatment (**Figure 4C**), suggesting that there was an association between these two proteins. To test whether there is a functional link between RFWD3 and Rad51 in our working system, we next established the cell line with interfered RFWD3 by its siRNA (**Figure 4D**, **Supplementary Figure 3D**). After disrupting RFWD3 in the transformed cells, Rad51 protein level in the combination group was increased, and its ubiquitination was decreased significantly as compared with IR alone (**Figures 4D, E**); whereas in the normal cells, the change of Rad51 protein level and its ubiquitination in the combination group was the opposite (**Figures 4D, E**). Thus, the data demonstrated RFWD3-dependent Rad51 ubiquitination was involved in the VPA-mediated bidirectional effect.

### VPA Targets RFWD3 for Bidirectional Regulation on HR Activity *In Vivo*

In our previous studies, the DMBA-induced breast tumor on female SD rats was demonstrated to be a stable primary breast tumor model (33, 46–48). Using this animal model (**Supplementary Figure 4A**), the function of RFWD3 was confirmed. We subjected the rat tumor model to a total of 8 Gy IR over 4 days (**Supplementary Figure 4B**, left panel). Some studies have reported that the recommended therapeutic equivalent doses of VPA for RT ranged from 150 to 600 mg/kg (31, 49). We have opted for 200 mg/kg (*i.p.*) VPA as this was the same as that used to treat the cells (0.5 mM) in our working system (**Supplementary Figure 4C**).

**Figure 5:**
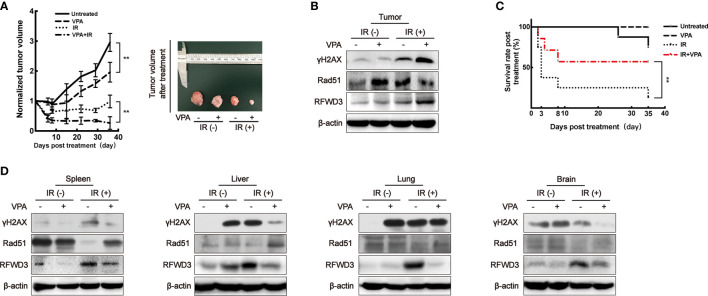
VPA targets RFWD3 for bidirectional regulation on HR activity *in vivo*. **(A)** Left panel: tumor volume of DMBA-induced primary breast cancer rats after treatment was normalized by the untreated group (n = 10 in each group). Right panel: tumors dissected from rats at the 35th day post-treatment. **(B)** Protein expression in tumor tissue was detected by specific antibodies through Western blot. **(C)** The survival rate of tumor rats following VPA and IR treatment (n = 10 in each group). **(D)** Proteins expression in normal tissues under VPA or IR were detected by specific antibodies through Western blot. Bands in the figures were quantified by ImageJ software (Wayne Rasband); each data point in the graph was from three independent experiments (mean ± SD); *P*-values were calculated by t-test (**P < 0.01).

When compared to the untreated group, the rate of tumor growth in the VPA alone group was slower (*P* < 0.01, **Figure 5A**). After the administration of both VPA and IR, tumor growth was significantly inhibited comparing to IR alone (*P* < 0.01, **Figure 5A**), the DSBs detected by γH2AX was enhanced (**Figure 5B**), the protein level of Rad51 was inhibited (**Figure 5B**), but RFWD3 protein level was increased (**Figure 5B**) as compared with IR treatment alone. The data concur with the above results obtained from the paired *in vitro* cell line. Our previous study uncovered that P53 can suppress the hyper-recombination by modulating BRCA1 and Rad51 (41), here we found that VPA increased the protein level of P53 and P21 in tumor tissue cells under IR treatment (**Supplementary Figure 4D**).

We observed that the survival rate of the rats with breast cancer was significantly decreased after IR treatment, this was ameliorated with VPA administration (*P*<0.01), thus implying a protective effect of VPA on the rats (**Figure 5C**). Therefore, we next investigated the status of abscopal normal tissues subjected to the *in vivo* treatments. After the combination treatment, the spleen, liver, lung, and brain tissues demonstrated that the levels of DSBs as detected by γH2AX were attenuated, while Rad51 protein levels were enhanced, and the protein levels of RFWD3 were decreased (**Figure 5D**), P53 and P21 protein expressions were also inhibited (**Supplementary Figure 4E**), as compared with IR treatment.

Thus, we demonstrated that the bidirectional effect of VPA *in vivo* through this animal tumor model during IR treatment was consistent with the findings from the *in vitro* cell lines. The VPA-mediated bidirectional effect was RFWD3-dependent and triggers Rad51-associated HR function.

### Higher Level of Rad51 in Human Breast Cancer May Be Associated With Poor Prognosis and Supported to be the Marker for Tumor Therapy

**Figure 6:**
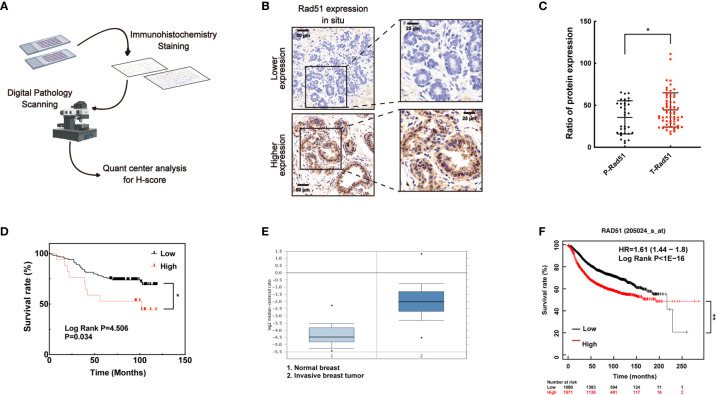
A higher level of Rad51 in human breast cancer may be associated with poor prognosis. **(A)** Human breast tumor tissue array was stained through immunohistochemistry by specific antibody, slides were digitally scanned and subjected to QuantCenter analysis. H-score was used to value the level of Rad51 expression. **(B)** Rad51 expression level in para-carcinoma tissues defined the standard of lower and higher level of Rad51 expression in breast tumor tissues. **(C)** The expression of Rad51 in para-carcinoma (P-Rad51, n = 35) and breast tumor (T-Rad51, n = 101) was counted in the graph. **(D)** The survival rate of patients who contributed the breast tumor tissue was calculated using the follow-up data of 120 months. **(E)** Rad51 expression level on a concomitant dataset was used to confirm the findings on tissue array. **(F)** Kaplan-Meier plotter (http://kmplot.com) was used to evaluate the association between prognosis and Rad51 expression. The number of patients who survived at different follow-up times was listed below the graph. Each data point in the graph was from three independent experiments (mean ± SD); *P*-values were calculated by t-test (*P < 0.05, **P < 0.01).

To investigate the association of Rad51 with the malignant carcinoma development and the significance of VPA in clinical application, IHC of Rad51 was performed in tissue arrays (**Figure 6A**) containing the samples of para-carcinoma breast tissues (n = 35) and breast tumor tissues (n = 79, **Figure 6B**). Rad51 expression in tumor tissues was higher than the expression in para-carcinoma tissues (*P* < 0.05, **Figure 6C**). Combining with the survival rate data for these patients, the results indicated that higher expression of Rad51 was associated with poor prognosis (*P* < 0.05, **Figure 6D**). This finding was triangulated with the concomitant dataset (*P* < 0.01; fold change, 4.969; n = 82, **Figure 6E**) and Kaplan-Meier plotter data set (*P* < 0.01; n = 3951; **Figure 6F**). The data suggested that Rad51 was involved in the oncogenic process and may serve as a significant marker and target of breast cancer in tumor therapy.

## Discussion

**Figure 7:**
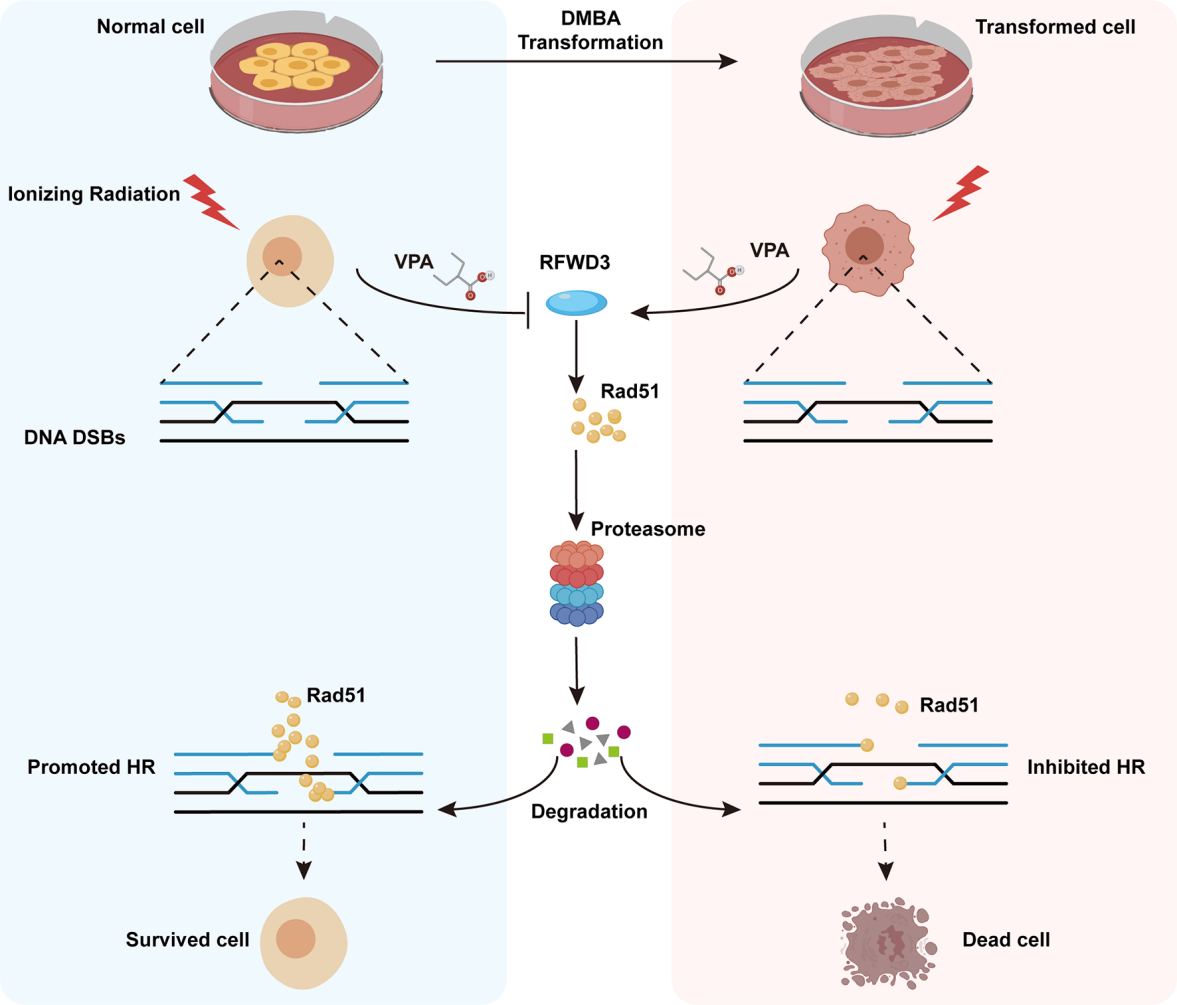
Working model illustrates the radiosensitizing and radioprotection effect of VPA. VPA sensitizes malignantly transformed cells while protects normal cells to IR treatment. VPA enhances the DSBs level in malignant cells and alleviates the DSBs level in normal cells through regulating HR function. RFWD3 is targeted by VPA to manipulate the ubiquitination of Rad51. VPA inhibites RFWD3 to decrease the ubiquitination of Rad51 in normal cells in response to IR-induced DSBs, protecting Rad51 from degradation, while the ubiquitination is enhanced in malignant cells.

Here, we have established a transformed cell model using normal MCF10A cells and the environmental carcinogen, DMBA, and verified its characteristics of malignancy. Using both the transformed and normal MCF10A cell lines, we confirmed the radiosensitization effect of VPA on malignant cells and discovered the radioprotective effect of VPA on normal breast cells and found a molecular mechanism for this bidirectional effect of VPA, which target E3 ligase RFWD3-dependent Rad51 ubiquitination. The findings were further validated by the well-established DMBA-induced breast cancer animal model. We also found that the high level of Rad51 was associated with invasive tumorigenesis and poorer prognosis in breast cancer patients. Thus, our findings uncovered the novel mechanism of bidirectional effect of VPA under IR treatment both *in vitro* and *in vivo*, VPA can directly target RFWD3 and then trigger Rad51 ubiquitination during cancer radiotherapy and limit radiotoxicity on normal cells (**Figure 7**).

### The Model of DMBA-Induced Transforming Cell and Animal Is Reliable for Tumor Study

Cell and animal-derived cancer models are usually established by the treatment of carcinogenic chemicals (50–52). DMBA as a prototypical polycyclic aromatic hydrocarbon (PAH) has been demonstrated to be carcinogenic with high cytotoxicity and immunotoxicity (42). Studies employed DMBA to establish multiple cancer models in animals, these include breast, oral, skin, and lung cancers (48, 53–55). DMBA is also used to induce the transformation of normal human breast epithelial cell MCF10A (42, 56). In this study, we established a stable transformed MCF10A cell line, and RNA-seq analysis found that breast cancer and other cancer pathways were activated, thus confirming the malignancy in this transformed cell line. In our previous studies, we have established the primary breast cancer model on female SD rats by DMBA (33). Both the DMBA-transformed cell and animal models effectively mimicked the tumorigenesis and the features of malignancy of human breast cancer. The successful establishment of these two models plays a great significance for the comparative study of tumor cells and normal cells in cancer prevention and treatment, as well as the study on the mechanism of tumorigenesis. Therefore, in the current study, the use of both models aims to triangulate the molecular mechanism of VPA-mediated bidirectional role in cancer radiotherapy.

### Up-Regulation of RFWD3-Dependent Rad51 Ubiquitination Plays a More Direct Role in VPA-Mediated Radiosensitization in Tumor Cells

As we have previously known, Rad51 plays a pivotal role in the HR process to repair damaged DNA. Studies from our and other groups suggested that VPA can suppress Rad51 activity for the radiosensitization to breast cancer cells (33, 57). Since Rad51 is an effector in the DNA damage signaling pathway, it would be necessary to explain how Rad51 protein level was down-regulated in the cellular process. In this study, we found that the decrease of Rad51 is associated with the increase of its ubiquitination in VPA-mediated radiosensitization for tumor cells in both the *in vivo* and *in vitro* working systems. The increase of Rad51 ubiquitination was caused by an E3 ligase, RFWD3. Therefore, VPA probably directly upregulates RFWD3 and then initiates Rad51 ubiquitination to disrupt the HR function so as to enhance IR effect for killing cancer cells. Some studies reported that the ubiquitination of DNA damage and repair-related proteins are important for keeping HR function (13, 19), RFWD3 as an E3 ligase can interact and ubiquitinate Rad51 to promote Rad51 timely removal from the damaged sites to regulate HR activity (18).

The NHEJ is another major pathway for DSBs repair in mammalian cells, which use little or no homologous template (58). DNA-PKcs and Ku proteins play a major role during NHEJ (59), however, the expression of these key proteins in NHEJ was not significantly altered following VPA and IR treatment in our current study, both in the transformed and normal cell lines, which is inconsistent with the results on MCF7 and U2OS cells (33, 57). Due to the characteristic difference and the phenotype between cell lines, the VPA-regulated effect may be different between cell lines, which requires further investigation.

### Down-regulation of RFWD3-Dependent Rad51 Ubiquitination Was Required for VPA-Mediated Radioprotection in Normal Cells

Since it was found VPA can radiosensitize tumor cells, it would be essential to understand how VPA influences normal cells during tumor RT. In the current study, VPA exhibited a radioprotective effect on normal cells both *in vivo* and *in vitro*, consistent with other studies, which reported VPA to be radioprotective in normal neuron cells (31, 32, 34), that total body IR-induced lethality of mice was mitigated by concomitant VPA (35), and VPA rescued the hair loss and enhanced the survival in high-grade glioma patients during RT (36). However, the mechanism of VPA-mediated radioprotection in normal cells is still unknown. In the present study, we found that the mechanism of VPA in normal cells was opposite to tumor cells, RFWD3-dependent Rad51 ubiquitination was down-regulated, which means that VPA-mediated radioprotection was associated with preserving Rad51 activity and avoiding its ubiquitination by RFWD3. Therefore, RFWD3 serves a different function in normal cells and tumor cells, and VPA can initiate the key switch of RFWD3-related ubiquitination to have a radio-bidirectional effect.

### The VPA-Mediated RFWD3-Dependent Bidirectional Effect Was Most Notable Upon IR Treatment

However, in the VPA alone treatment, we found that RFWD3 was upregulated compared to the untreated group in the transformed cells, and the Rad51 expression was slightly downregulated while the ubiquitination of Rad51 was upregulated marginally. Correspondingly, in the normal cells, RFWD3 was inhibited following the treatment of VPA alone, the ubiquitination of Rad51 was marginally affected, while the expression of Rad51 did not enhance but was preserved at a similar level as the untreated group. Thus, we speculate that this VPA-mediated RFWD3-dependent regulation effect was most notably by IR-induced high-level of ubiquitination.

### Lateral Pathways May Potentially Involve in the VPA-Mediated Radio-Bidirectional Effect

Some studies demonstrated that VPA can enhance the efficiency of chemotherapy and radiotherapy by affecting apoptosis and cell cycle pathways (60, 61). We explored the dominant role of Rad51 ubiquitination in apoptosis by detecting the biological phenotypes under inhibiting the observed apoptosis and the RFWD3-mediated Rad51 ubiquitination function (**Supplementary Figure 5A**). Employing the pan-caspase inhibitor, Z-VAD-FMK, which can inhibit and reverse the apoptotic event (62), we found that the effect of VPA on Rad51 ubiquitination was not affected both in the transformed and normal MCF10A cells (**Supplementary Figures 5A, B**). Importantly, si-RFWD3 transfection caused inhibition of Rad51 ubiquitination compromised the VPA-mediated bidirectional effect upon IR treatment in these two cells (**Supplementary Figure 5C**). The data suggested that Rad51 ubiquitination was prior to apoptosis.

Our previous studies indicated that VPA can sensitize breast cancer cells to chemotherapy and IR treatments by inhibiting the HR function, such VPA-mediated HR inhibition was not associated with the cell cycle (47, 63). Since we have employed the same treatments (0.5 mM VPA and 8 Gy IR) in the present working system, therefore the cell cycle would not be involved into the VPA-mediated bidirectional effect.

Due to the complicated intracellular functions during DDR, there may be multiple modification pathways involving in this VPA-mediated radio-bidirectional effect, which required further investigation.

### VPA Shows Perspective Application on Clinical RT

At present, the effectiveness of RT is limited by the toxicity to normal cells during IR treatment, hence locating a safe and efficacious adjuvant treatment to alleviate IR-induced damage in normal cells while sensitizing tumor cells to IR has attracted much clinical attention. VPA is a potential candidate with its dual radioprotective and radiosensitizing effects (64, 65). We believe that the findings of this radio-bidirectional effect of VPA and its molecular mechanism will spur further investigation into VPA’s clinical application in cancer therapy.

## Data Availability Statement

The original contributions presented in the study are included in the article/**Supplementary Material**. Further inquiries can be directed to the corresponding author.

## Ethics Statement

The studies involving human participants were reviewed and approved by Human Research Ethics Committee (HREC) of Taizhou hospital of Zhejiang Province. The patients/participants provided their written informed consent to participate in this study. The animal study was reviewed and approved by Shandong University Human and Animal Ethics Research Committee.

## Author Contributions

GL: Research conceptualization, data collection and analysis, writing of manuscript; DL, CD, FZ, GG, XW, PZ: data validation, review drafting of manuscript; ZC, WD, ZT: data validation; ZF: Research conceptualization, data analysis, review and drafting of manuscript, project supervision, project administration, funding acquisition. All authors contributed to the article and approved the submitted version.

## Funding

This project was supported by grants from the National Natural Science Foundation of China (81472800), the Key Technology Research and Development Program of Shandong (2019GSF108083), and the Natural Science Foundation of Shandong Province (ZR2020MH330).

## Conflict of Interest

The authors declare that the research was conducted in the absence of any commercial or financial relationships that could be construed as a potential conflict of interest.

## Supplementary Material

Supplementary Figure 1
**(A)** Normal cells (left panel) and transformed cells (right panel) were pretreated with VPA for 24h and subjected to 8Gy IR. The DNA DSBs level of cells at 0min, 30min, and 120min post-IR were detected by **(A)** alkaline comet assay. By immunofluorescence, **(B)** 53BP1 foci formation in the paired cells was presented. The percentage of L-type and H-type of the cells containing 53BP1 foci was showed the graph. The survival of **(C)** 16HBE cell was detected by colony formation assay. The cell survival fraction in the difference between groups was shown in the graph. At the time of 6h post-IR, **(D)** normal, transformed MCF10A and **(E)** breast tumor cell lines MCF7, MDA-MB-231, EUFA423 and normal 16HBE cells were subjected to western blotting. **(F)** At the time of the 6th and 24th hour, treated cells were delivered to immunofluorescence assay. BRCA1 foci formation was showed in the pictures, columns in the graphs presented the cell percentage expressed protein foci. Each data point in the graph was from three independent experiments (mean ± SD); *P*-values were calculated by t-test (**P*<0.05, ** *P*<0.01).

Supplementary Figure 2
**(A)** 10μM RI-1 inhibited the expression of Rad51 protein after 24h- pretreatment with VPA before 8Gy IR treatment. **(B)** RI-1 working system was performed on 16HBE ells. Colony formation assay on 16HBE cells showed the survival fraction and the quantification graph was showed in figure. **(C)** The stable expressed cell line of BRCA1-deficient and BRCA1-proficient was successfully established by the BRCA1-deficient cell line, HCC1937. By western blot, the BRCA1 protein level was detected in the isogenic paired cells. MCF7 was used as a positive control of BRCA1 expression. By colony formation assay, cell survival in the paired cell lines was performed after different treatments. Each data point in the graphs was from three independent experiments (mean ± SD); *P*-values were calculated by t-test (** *P*<0.01).

Supplementary Figure 3 After VPA and IR treatments, **(A)** transformed and **(B)** normal MCF10A cells were treated with 40μg/ml CHX for 0h, 2h, 4h, 8h. The expression of BRCA1 was detected by western blot. **(C)** RFWD3 protein expression was detected in MCF7, MDA-MB-231, EUFA423, 16HBE cells following VPA and IR treatments. **(D)** siRNA was designed to abolish the function of RFWD3 and the expression of Rad51 under those treatments was detected by western blot. Notes: bands in the graphs were quantified by ImageJ software (Wayne Rasband). Each data point in the graphs was from three independent experiments (mean ± SD); *P*-values were calculated by t-test (** *P*<0.01).

Supplementary Figure 4
**(A)** Primary breast tumor model was induced by environmental carcinogen DMBA. The schematic diagram of the distribution of normal breast (left upper panel) and DMBA-induced primary breast tumor (left lower panel) is shown in figure. The pathological morphology of this primary tumor model is exhibited (right panel). **(B)** The schedule of VPA administration and 2Gy ×4 days of IR treatment. VPA was injected into rats every 12h. VPA injection was performed before and after the daily 2Gy irradiation, total 12 times of VPA injection (left panel). Breast tumors on rats were pulled to a side of the body to avoid direct irradiation on normal tissues, the irradiation field was adjusted to the size of the tumors to ensure accurate targeting on tumor (right panel). **(C)** The dynamic concentration of VPA in serum was detected by a pharmacokinetic assay on SD rats. VPA solution at different concentrations were delivered to rats by gavage (*i.g.*) or intraperitoneal injection (*i.p.*). Whole blood from the jugular vein at time points was centrifuged for the VPA detection in serum. (D-E) proteins expression were detected by specific antibodies through western blot. Notes: each data point in the graph was from three independent experiments (mean SD).

Supplementary Figure 5
**(A)** The protein expression of total caspase3, cleaved caspase3 and RFWD3 were detected under VPA and IR treatment or the inhibition of caspase-mediated apoptosis by Z-VAD-FMK and the transfection of si-RFWD3 both in transformed and normal MCF10A. **(B)** The comparison of the VPA+IR and the IR alone group under si-RFWD3 or not in transformed and normal MCF10A through quantified intensity of protein bands. **(C)** The ubiquitination of Rad51 in those two cells were detected under VPA and IR treatment or the inhibition of caspase-mediated apoptosis by Z-VAD-FMK and the transfection of si-RFWD3. Notes: bands in the figures were quantified by ImageJ software (Wayne Rasband); each data point in the graph was from three independent experiments (mean ± SD); *P*-values were calculated by t-test (** *P*<0.01, NS: no significant differences).
