# Supplementary figures and images for: The Valproate Mediates Radio-Bidirectional Regulation Through RFWD3-Dependent Ubiquitination on Rad51
Source: Front Oncol. 2021 Mar 25;11:646256. doi: 10.3389/fonc.2021.646256 (PMC8029989; doi:10.3389/fonc.2021.646256)

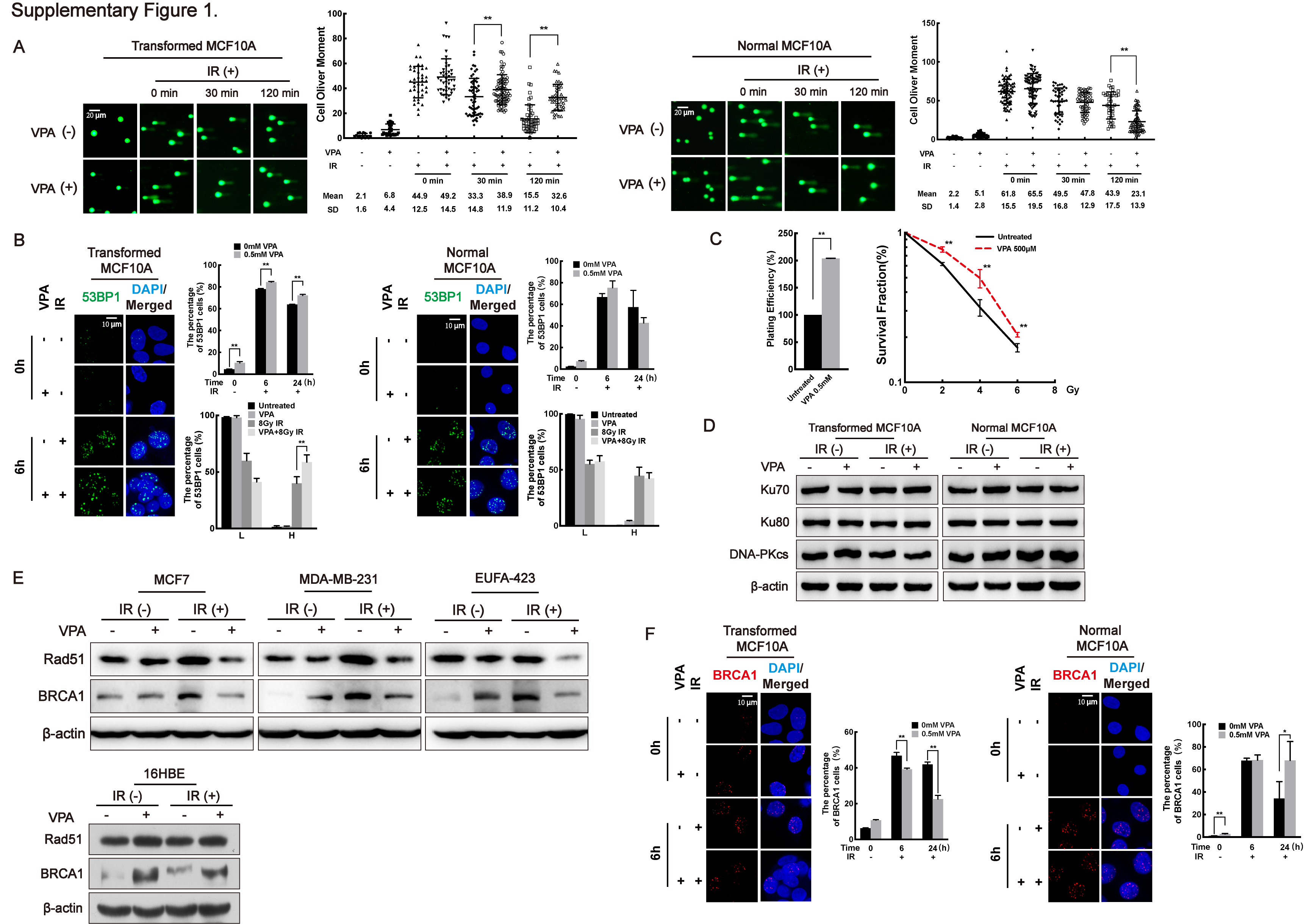

Supplement: Supplementary Figure 1 — (A) Normal cells (left panel) and transformed cells (right panel) were pretreated with VPA for 24h and subjected to 8Gy IR. The DNA DSBs level of cells at 0min, 30min, and 120min post-IR were detected by (A) alkaline comet assay. By immunofluorescence, (B) 53BP1 foci formation in the paired cells was presented. The percentage of L-type and H-type of the cells containing 53BP1 foci was showed the graph. The survival of (C) 16HBE cell was detected by colony formation assay. The cell survival fraction in the difference between groups was shown in the graph. At the time of 6h post-IR, (D) normal, transformed MCF10A and (E) breast tumor cell lines MCF7, MDA-MB-231, EUFA423 and normal 16HBE cells were subjected to western blotting. (F) At the time of the 6th and 24th hour, treated cells were delivered to immunofluorescence assay. BRCA1 foci formation was showed in the pictures, columns in the graphs presented the cell percentage expressed protein foci. Each data point in the graph was from three independent experiments (mean ± SD); P-values were calculated by t-test (*P<0.05, ** P<0.01). [file Image_1.tif]

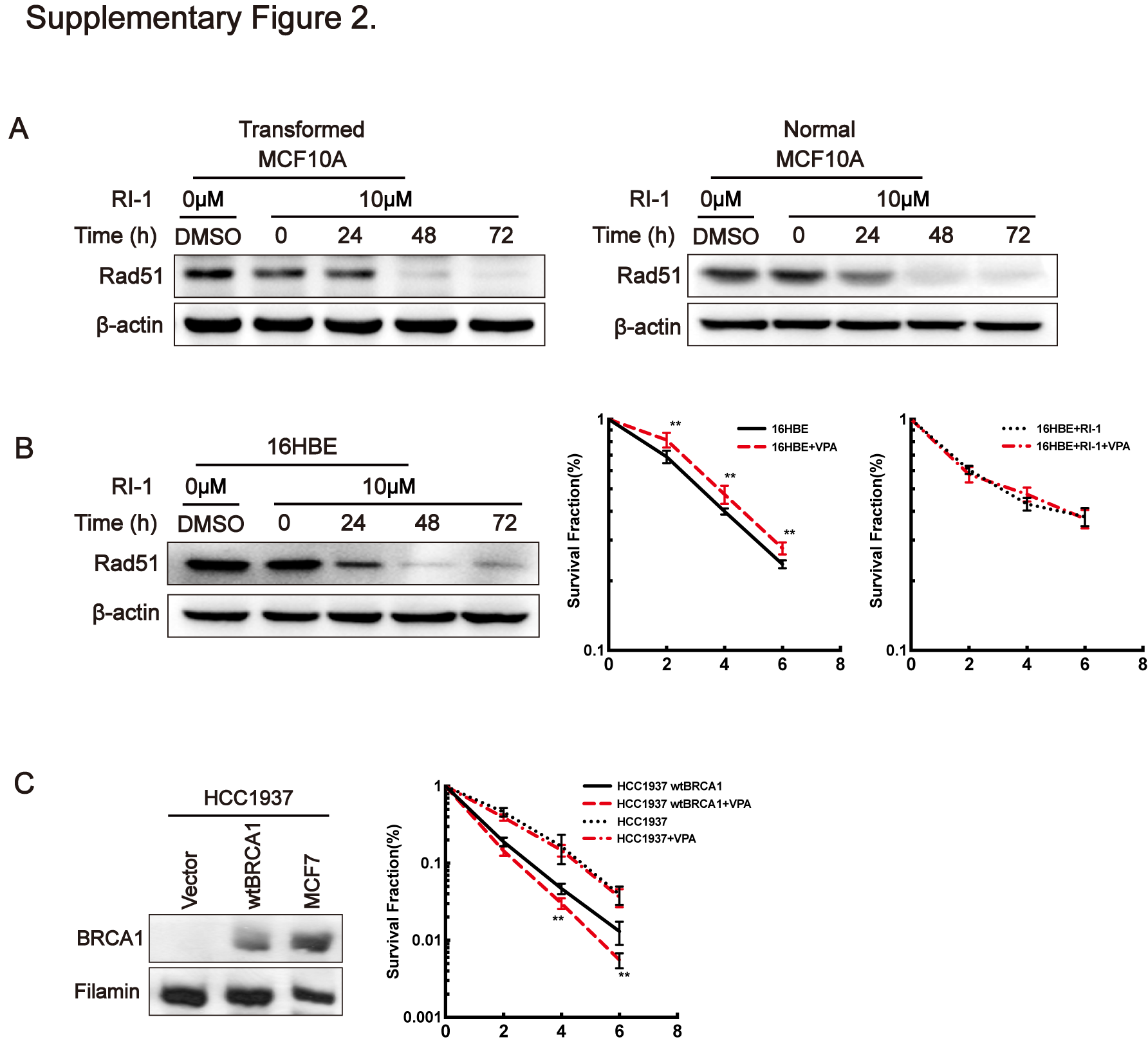

Supplement: Supplementary Figure 2 — (A) 10μM RI-1 inhibited the expression of Rad51 protein after 24h- pretreatment with VPA before 8Gy IR treatment. (B) RI-1 working system was performed on 16HBE ells. Colony formation assay on 16HBE cells showed the survival fraction and the quantification graph was showed in figure. (C) The stable expressed cell line of BRCA1-deficient and BRCA1-proficient was successfully established by the BRCA1-deficient cell line, HCC1937. By western blot, the BRCA1 protein level was detected in the isogenic paired cells. MCF7 was used as a positive control of BRCA1 expression. By colony formation assay, cell survival in the paired cell lines was performed after different treatments. Each data point in the graphs was from three independent experiments (mean ± SD); P-values were calculated by t-test (** P<0.01). [file Image_2.tif]

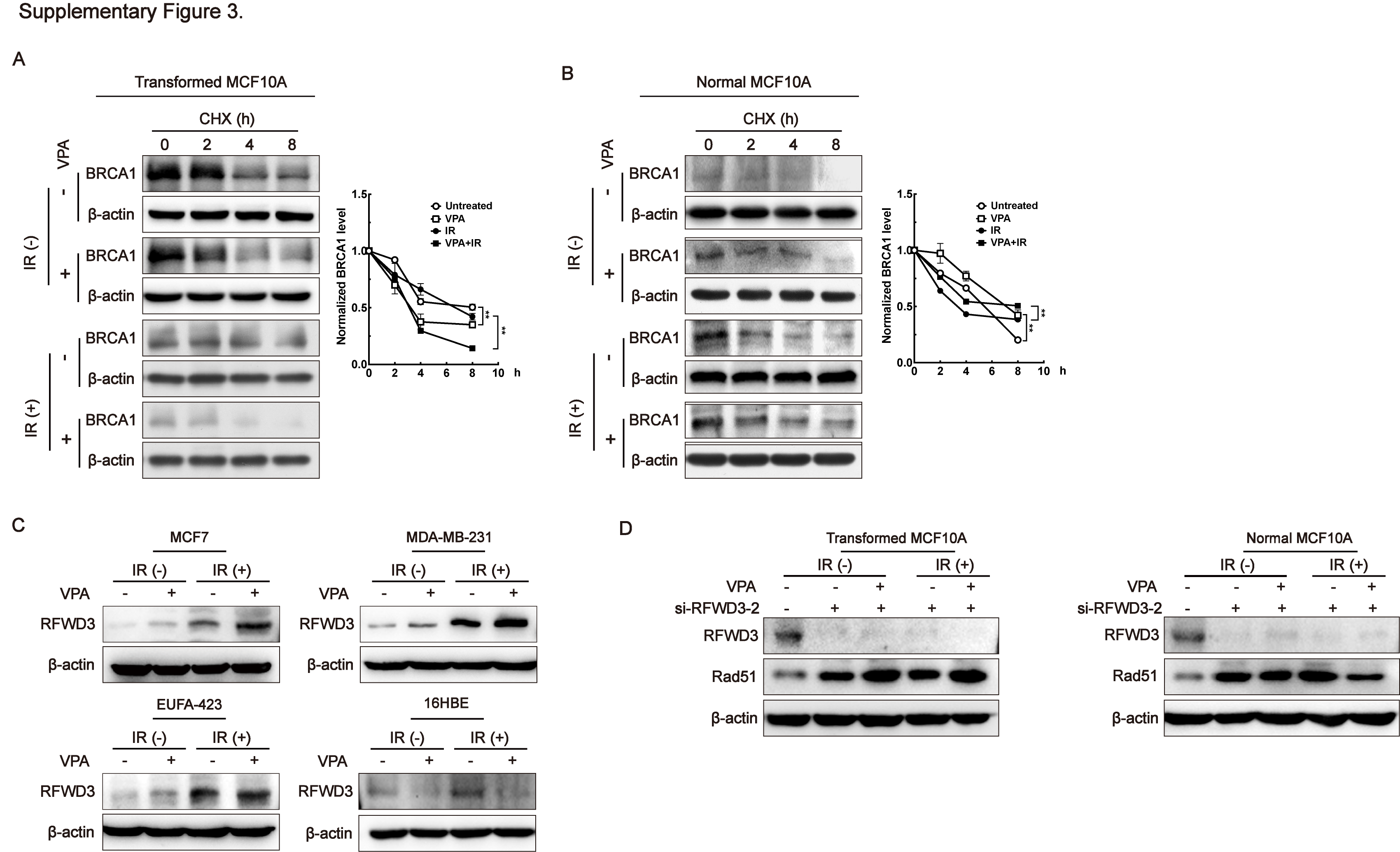

Supplement: Supplementary Figure 3 — After VPA and IR treatments, (A) transformed and (B) normal MCF10A cells were treated with 40μg/ml CHX for 0h, 2h, 4h, 8h. The expression of BRCA1 was detected by western blot. (C) RFWD3 protein expression was detected in MCF7, MDA-MB-231, EUFA423, 16HBE cells following VPA and IR treatments. (D) siRNA was designed to abolish the function of RFWD3 and the expression of Rad51 under those treatments was detected by western blot. Notes: bands in the graphs were quantified by ImageJ software (Wayne Rasband). Each data point in the graphs was from three independent experiments (mean ± SD); P-values were calculated by t-test (** P<0.01). [file Image_3.tif]

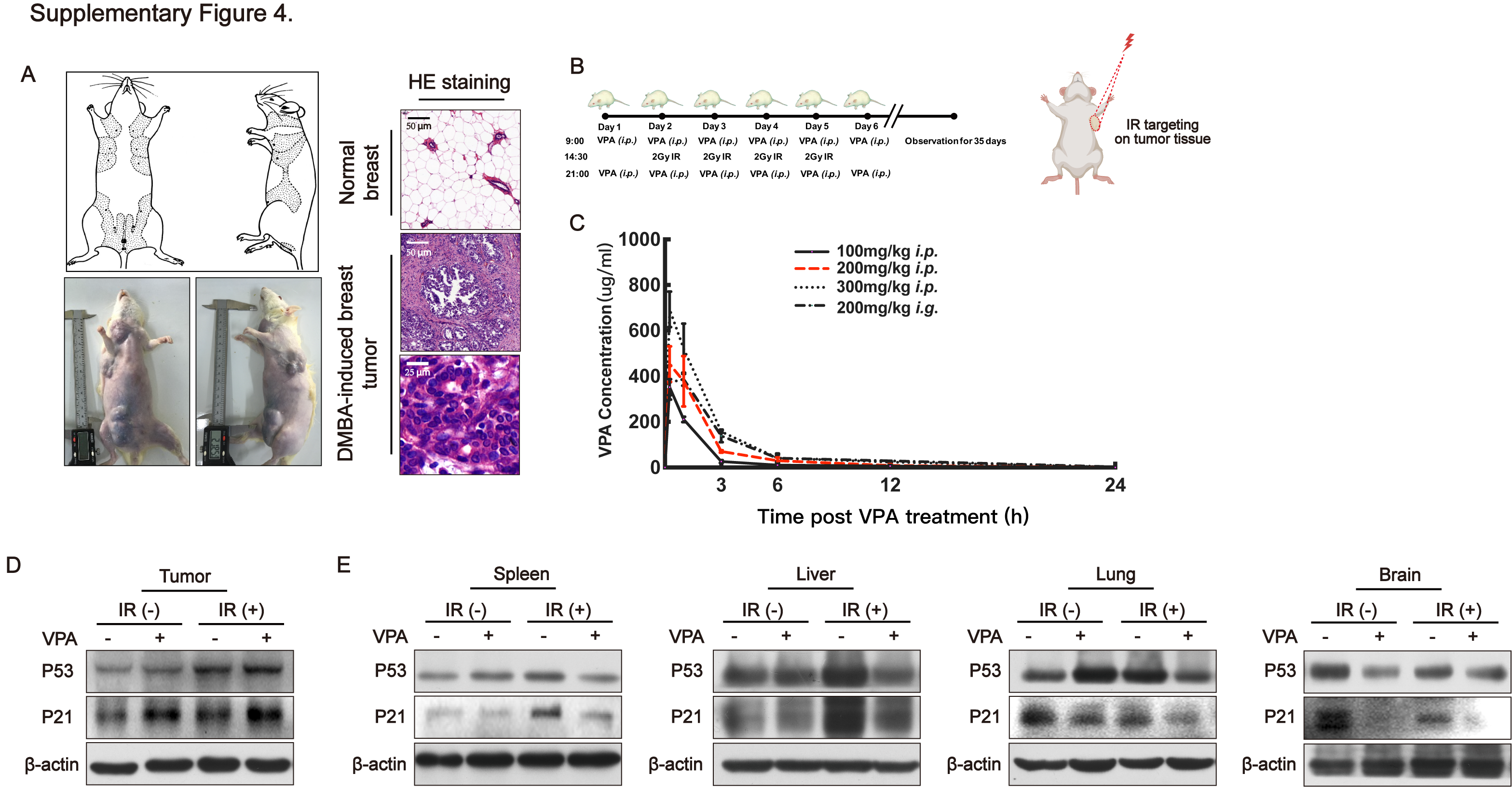

Supplement: Supplementary Figure 4 — (A) Primary breast tumor model was induced by environmental carcinogen DMBA. The schematic diagram of the distribution of normal breast (left upper panel) and DMBA-induced primary breast tumor (left lower panel) is shown in figure. The pathological morphology of this primary tumor model is exhibited (right panel). (B) The schedule of VPA administration and 2Gy ×4 days of IR treatment. VPA was injected into rats every 12h. VPA injection was performed before and after the daily 2Gy irradiation, total 12 times of VPA injection (left panel). Breast tumors on rats were pulled to a side of the body to avoid direct irradiation on normal tissues, the irradiation field was adjusted to the size of the tumors to ensure accurate targeting on tumor (right panel). (C) The dynamic concentration of VPA in serum was detected by a pharmacokinetic assay on SD rats. VPA solution at different concentrations were delivered to rats by gavage (i.g.) or intraperitoneal injection (i.p.). Whole blood from the jugular vein at time points was centrifuged for the VPA detection in serum. (D-E) proteins expression were detected by specific antibodies through western blot. Notes: each data point in the graph was from three independent experiments (mean SD). [file Image_4.tif]

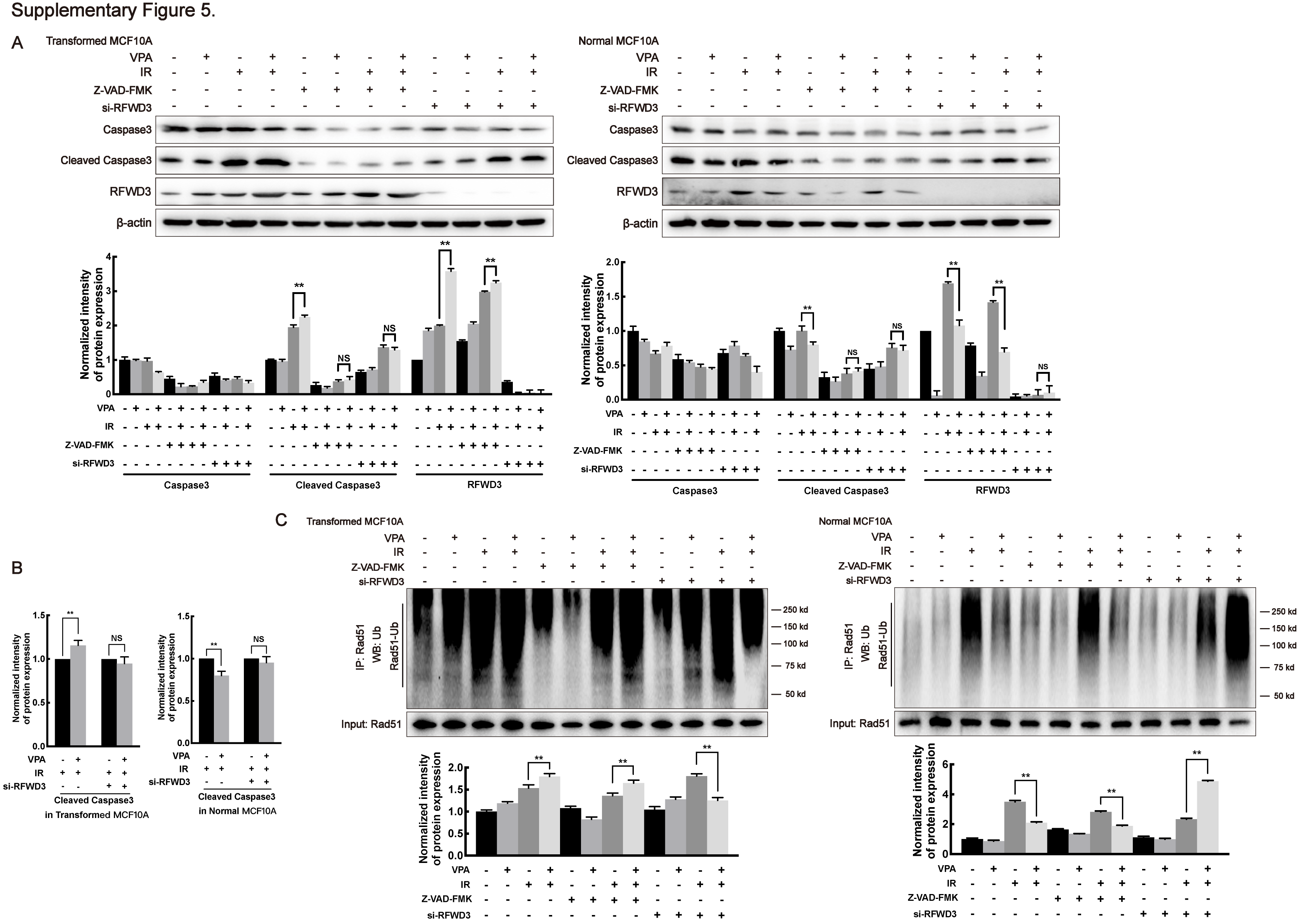

Supplement: Supplementary Figure 5 — (A) The protein expression of total caspase3, cleaved caspase3 and RFWD3 were detected under VPA and IR treatment or the inhibition of caspase-mediated apoptosis by Z-VAD-FMK and the transfection of si-RFWD3 both in transformed and normal MCF10A. (B) The comparison of the VPA+IR and the IR alone group under si-RFWD3 or not in transformed and normal MCF10A through quantified intensity of protein bands. (C) The ubiquitination of Rad51 in those two cells were detected under VPA and IR treatment or the inhibition of caspase-mediated apoptosis by Z-VAD-FMK and the transfection of si-RFWD3. Notes: bands in the figures were quantified by ImageJ software (Wayne Rasband); each data point in the graph was from three independent experiments (mean ± SD); P-values were calculated by t-test (** P<0.01, NS: no significant differences). [file Image_5.tif]
